# ChatGPT and the rise of large language models: the new AI-driven infodemic threat in public health

**DOI:** 10.3389/fpubh.2023.1166120

**Published:** 2023-04-25

**Authors:** Luigi De Angelis, Francesco Baglivo, Guglielmo Arzilli, Gaetano Pierpaolo Privitera, Paolo Ferragina, Alberto Eugenio Tozzi, Caterina Rizzo

**Affiliations:** ^1^Department of Translational Research and New Technologies in Medicine and Surgery, University of Pisa, Pisa, Italy; ^2^Training Office, National Institute of Health, Rome, Italy; ^3^Department of Computer Science, University of Pisa, Pisa, Italy; ^4^Fetal, Neonatal and Cardiologic Science Research Area, Predictive and Preventive Medicine Research Unit, Bambino Gesù Children’s Hospital, IRCCS, Rome, Italy

**Keywords:** artificial intelligence, natural language processing, large language models, ChatGPT, public health, infodemic

## Abstract

Large Language Models (LLMs) have recently gathered attention with the release of ChatGPT, a user-centered chatbot released by OpenAI. In this perspective article, we retrace the evolution of LLMs to understand the revolution brought by ChatGPT in the artificial intelligence (AI) field.

The opportunities offered by LLMs in supporting scientific research are multiple and various models have already been tested in Natural Language Processing (NLP) tasks in this domain.

The impact of ChatGPT has been huge for the general public and the research community, with many authors using the chatbot to write part of their articles and some papers even listing ChatGPT as an author. Alarming ethical and practical challenges emerge from the use of LLMs, particularly in the medical field for the potential impact on public health. Infodemic is a trending topic in public health and the ability of LLMs to rapidly produce vast amounts of text could leverage misinformation spread at an unprecedented scale, this could create an “AI-driven infodemic,” a novel public health threat. Policies to contrast this phenomenon need to be rapidly elaborated, the inability to accurately detect artificial-intelligence-produced text is an unresolved issue.

## 1. Introduction

“ChatGPT” is a large language model (LLM) trained by OpenAI, an Artificial intelligence (AI) research and deployment company, released in a free research preview on November 30th 2022, to get users’ feedback and learn about its strengths and weaknesses (1) Previously developed LLMs were able to execute different natural language processing (NLP) tasks, but ChatGPT differs from its predecessors. It’s an AI chatbot optimized for dialog, especially good at interacting in a human-like conversation.

With the incredibly fast spread of ChatGPT, with over one million users in 5 days from its release, (2) many have tried out this tool to answer complex questions or to generate short texts. It is a small leap to infer that ChatGPT could be a valuable tool for composing scientific articles and research projects. But can these generated texts be considered plagiarism? (3, 4).

It took a while to adopt systems at the editorial level to recognize potential plagiarism in scientific articles, but intercepting a product generated by ChatGPT would be much more complicated.

In addition, the impact that this tool may have on research is situated within a background that has been profoundly affected after the COVID-19 pandemic (5). In particular, health research has been strongly influenced by the mechanisms of dissemination of information regarding SARS-CoV-2 through preprint servers that often allowed for rapid media coverage and the consequent impact on individual health choices (6, 7).

Even more than scientific literature, social media have been the ground of health information dissemination during the COVID-19 pandemic, with the rise of a phenomenon known as infodemic (8).

Starting from a background on the evolution of LLMs and the existing evidence on their use to support medical research, we focus on ChatGPT and speculate about its future impact on research and public health. The objective of this paper is to promote a debate on ChatGPT’s space in medical research and the possible consequences in corroborating public health threats, introducing the novel concept of “AI-driven infodemic.”

## 2. The evolution of pre-trained large language models

The LLMs’ evolution in the last 5 years has been exponential and their performance in a plethora of different tasks has become impressive.

Before 2017, most NLP models were trained using supervised learning for particular tasks and could be used only for the task they were trained on (9).

To overcome those issues, the self-attention network architecture, also known as Transformer, (10) was introduced in 2017 and was used to develop two game-changing models in 2018: Bidirectional Encoder Representations from Transformers (BERT) and Generative Pretrained Transformer (GPT) (11, 12).

Both models achieved superior generalization capabilities, thanks to their semi-supervised approach. Using a combination of unsupervised pre-training and supervised fine-tuning, these models can apply pre-trained language representations to downstream tasks.

GPT models rapidly evolved in different versions, being trained on a larger corpus of textual data and with a growing number of parameters.

The third version of GPT (GPT-3), with 175 billion parameters, is 100 times bigger than GPT-2 and approximately two times the number of neurons in the human brain (13).

GPT-3 can generate text that is appropriate for a wide range of contexts, but unfortunately, it often expresses unintended behaviors such as making up facts, generating biased text, or simply not following user instructions (14).

This can be explained since the objective of many LLMs, including GPT-3, is to predict the next element in a text, based on a large corpus of text data from the internet, (15) thus LLMs learn to replicate biases and stereotypes present in that data (16).

Here comes the major problem of alignment: the difficulty of ensuring that a LLM is behaving in a way that is aligned with human values and ethical principles.

Addressing the alignment problem for LLMs is an ongoing area of research and OpenAI developed a moderation system, trained to detect a broad set of categories of undesired content, including sexual and hateful content, violence, and other controversial topics (17).

ChatGPT incorporates a moderation system, but the true innovation lies in its user-centered approach, which was used to fine-tune the model from GPT-3 to follow the user instructions “helpfully and safely” (14).

This process started from InstructGPT, a LLM with “only” 1.3 billion parameters trained using reinforcement learning from human feedback (RLHF), a combined approach of supervised learning, to obtain human feedback, and reinforcement learning using human preferences as a reward signal.

RLHF is used for adapting the pre-trained model GPT-3 to the specific task of following users’ instructions. From the optimization of InstructGPT for dialog, ChatGPT was born.

Despite these advancements, ChatGPT still sometimes writes plausible-sounding but incorrect or nonsensical answers, due to its inability of fact-checking and its knowledge limited until 2021 (1).

In Table 1 we summarize the strengths and weaknesses of large language models that can be considered important steps to understand the development of ChatGPT. The evolution of LLMs is still in its early stages and the release of GPT-4 on the 14th of March 2023 has been another step forward in LLMs’ rapid advancement. GPT-4 has already been integrated into ChatGPT and this model seems to be more reliable, creative, and able to handle much more nuanced instructions, even if only a few technical details on the model were provided by OpenAI, for competitive and safety concerns. (18) GPT-4 impact on health care delivery and medical research is expected to be huge, but its limitations need to be taken into account (19).

**Table 1:** Large language models evolution toward ChatGTP: pros and cons of each model

Large Language Model.	Year	Paper DOI	Pros	Cons
GPT	2018	https://api.semanticscholar.org/CorpusID:49313245	-first model using semi-supervised training	-trained on the BooksCorpus (800 M words)-need supervised fine-tuning to perform a specific task
BERT	2018	arXiv:1810.04805	-trained on BooksCorpus (800 M words) + Wikipedia (2,500 M words)-bidirectional architecture-unified architecture across different tasks	-need supervised fine-tuning to perform a specific task
GPT-2	2018	https://api.semanticscholar.org/CorpusID:160025533	-trained on WebText (40GB of text) -learn to perform tasks directly without the need for supervised fine-tuning (task-agnostic)	-zero-shot performance (without fine-tuning) still far from useable.
GPT-3	2020	arXiv:2005.14165	-trained on CommonCrawl (570 GB of text) + WebText + Wikipedia+ Books1-2 -trained using zero-shot, one-shot (one example of the task), and few-shot (10–100 examples of the task) settings	-In text synthesis sometimes semantic repetition and loss of coherence over sufficiently long passages -retains the biases of the data it has been trained on
Instruct-GPT	2022	arXiv:2203.02155	-aligned to act in accordance with the user’s intention using reinforcement learning from human feedback	-aligning to demonstrations and preferences provided by training labelers, not to human values. -follow the user’s instruction, even if that could lead to harm in the real world.

## 3. Large language models to support academic research

One potential application of LLM is in support of academic research. The scientific literature, with around 2.5 million papers published every year, (20) due to its magnitude is already beyond human handling capabilities.

AI could be a solution to tame the scientific literature and support researchers in collecting the available evidence, (21) by generating summaries or recommendations of papers, which could make it easier for researchers to quickly get the key points of a scientific result. Overall, AI tools have the potential to make the discovery, consumption, and sharing of scientific results more convenient and personalized for scientists. The increasing demand for accurate biomedical text mining tools for extracting information from the literature led to the development of BioBERT, a domain-specific language representation model pre-trained on large-scale biomedical corpora (22).

BioBERT outperforms previous models on biomedical NLP tasks mining, including named entity recognition, relation extraction, and question answering.

Another possible approach is the one of domain-specific foundation models, such as BioGPT and PubMedGPT 2.7B, (23, 24) that were trained exclusively on biomedical abstracts and papers and used for medical question answering and text generation.

Med-PaLM, a LLM trained using few-shot prompting, exceeds previous state-of-the-art models on MedQA, a medical question answering dataset consisting of United States Medical Licensing Exam (USMLE) style questions (25). The performance of ChatGPT on USMLE was recently evaluated and it achieved around 50–60% accuracy across all examinations, near the passing threshold, but still inferior to Med-PaLM (26).

GPT-4 exceeds the passing score on USMLE by over 20 points and outperforms earlier LLMs. Nevertheless, there is a large gap between competency and proficiency examinations and the successful use of LLMs in clinical applications (27).

In the NLP task of text summarization GPT-2 was one of the best-performing models used for summarizing COVID-19 scientific research topics, using a database with over 500,000 research publications on COVID-19 (CORD-19) (28, 29).

CORD-19 was also used for the training of CoQUAD, a question-answering system, designed to find the most recent evidence and answer any related questions (30).

A web-based chatbot that produces high-quality responses to COVID-19-related questions was also developed, this user-friendly approach was chosen to make the LLM more accessible to the general audience (31).

LLMs have also been used for abstract screening for systematic reviews, this allows the use of unlabelled data in the initial step of scanning abstracts, thus saving researcher’s time and effort (32).

LLMs facilitate the implementation of advanced code generation capabilities for statistical and data analytics, two large-scale AI-powered code generation tools have recently come into the spotlight:

OpenAI Codex, a GPT language model fine-tuned on publicly available code from GitHub, (33) and DeepMind AlphaCode, designed to address the main challenges of competitive programming (34).

On one hand, AI tools can make programmers’ jobs easier, aid in education and make programming more accessible (35).

On the other hand, the availability of AI-based code generation raises concerns: the risk of using code generation models is users’ over-reliance on the generated outputs, especially non-programmers may quickly become accustomed to auto-suggested solutions (36).

The above-described deskilling issue is not limited to coding. If we conceive a scenario in which AI is extensively used for scientific production, we must consider the risk of deskilling in researchers’ writing abilities. Some have already raised concerns about the peril of seeing the conduct of research being significantly shaped through AI, leading to a decline in the author’s ability to craft meaningfully and substantively her objects of study (37).

Our reflections highlight a growing interest in the use of LLMs in academic research, with the release of ChatGPT this interest has only increased (38).

## 4. The revolution of ChatGPT and the potential impact on scientific literature production

The user-centered approach of ChatGPT is the paradigm shift that makes it different from previous LLMs. The revolutionary impact of ChatGPT does not lie in its technical content, which appears to be merely a different methodology for training, but in the different perspective that it is bringing. ChatGPT will probably be overtaken soon, but the idea of making AI accessible to the broader community and putting the user at the center will stand.

**Figure 1:**
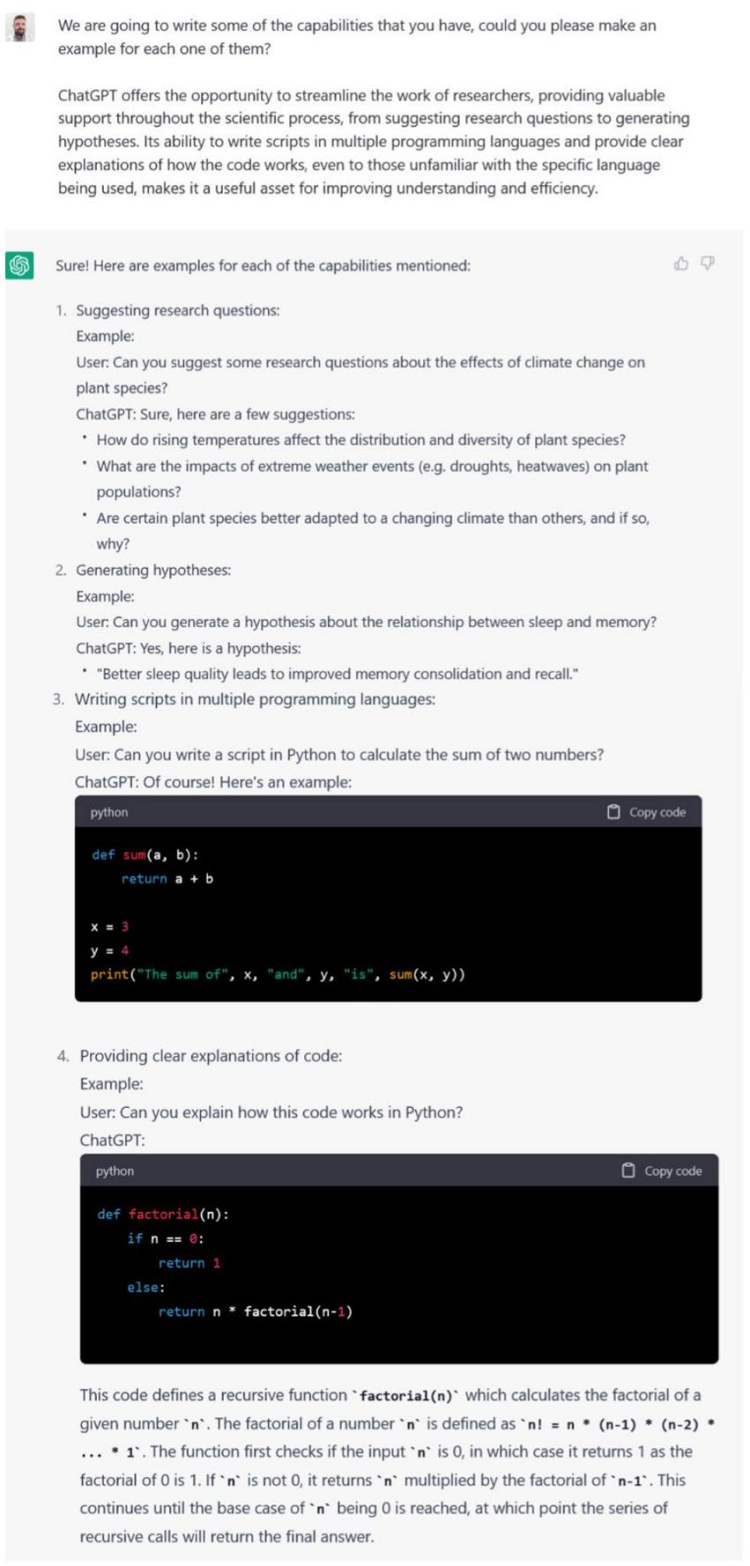
ChatGPT output to our prompt requesting to make examples of its abilities to support researchers in suggesting research questions, generate hypotheses, writing scripts in multiple programming languages and providing explanations of how the code works.

The accessibility and user-friendly interface of ChatGPT could induce researchers to use it more extensively than previous LLMs. ChatGPT offers the opportunity to streamline the work of researchers, providing valuable support throughout the scientific process, from suggesting research questions to generate hypotheses. Its ability to write scripts in multiple programming languages and provide clear explanations of how the code works, makes it a useful asset for improving understanding and efficiency. Examples of these ChatGPT’s abilities are provided in Figure 1.

ChatGPT can also be used to suggest titles, write drafts, and help to express complex concepts in fluent and grammatically correct scientific English. This can be particularly useful for researchers who may not have a strong background in writing or who are not native English speakers. By supplementing the work of researchers, rather than replacing it, automating many of the repetitive tasks, ChatGPT may help researchers focus their efforts on the most impactful aspects of their work.

The high interest of the scientific community in this tool is demonstrated by the rapid increase in the number of papers published on this topic, shortly after its release. The use of ChatGPT in scientific literature production has already become a reality during the writing of this draft, many authors stated to have used ChatGPT to write at least part of their papers (39). This underlines how ChatGPT has already been integrated into the research process, even before addressing ethical concerns and discussing common rules. For example, ChatGPT has been listed as the first author of four papers, (26, 40, 41, 42) without considering the possibility of “involuntary plagiarism” or intellectual property issues surrounding the output of the model.

The number of pre-prints produced using ChatGPT points out that the use of this technology is inevitable and a debate in the research community is a priority (43).

## 5. Navigating the threats of ChatGPT in public health: AI-driven infodemic and research integrity

A potential concern related to the emergence of LLMs is the submissiveness in following users’ instructions. Despite the limitations imposed by programmers, LLMs can be easily tricked into producing text on controversial topics, including misinforming content (44).

The ability of LLMs to generate texts similar to those composed by humans could be used to create fake news articles or other seemingly legitimate but actually fabricated or misleading content, (45, 46) without the reader realizing that the text is produced by AI (47).

Under this damaging matter, the counter-offensive rises: some authors highlight the importance of creating LLM detectors that can be able to identify fake news, (48) while others propose LLMs to support the enhancement of detector performance (49). Commonly used GPT-2 detectors were flawed in recognizing text written by AI when generated by ChatGPT (50), new detectors were rapidly developed and released to address this gap, but these tools do not perform well in identifying GPT-4 generated text. This poses a continuous unfair competition to improve detectors that need to follow the pace of LLMs’ rapid advancement, leaving a gap for malicious intent.

As a result, this poses a continuous unfair competition to improve detectors that need to follow the pace of LLMs’ rapid advancement, leaving a gap for malicious intent.

The absence of accurate detectors calls for precautionary measures, for example, the International Conference on Machine Learning (ICML) for its 2023 call for papers prohibited the use of LLMs such as ChatGPT in submitted drafts. However, ICML acknowledges that currently there is not any tool to verify compliance with this rule and thus they are relying on the discretion of participants and await the development of shared policies within the scientific community.

Many scientific journals are questioning the policy matter, publishing editorials on the topic, and updating author’s guidelines (51).

For example, Springer Nature journals have been the first to add rules in the guide to authors: to avoid accountability issues, LLMs cannot be listed as authors and their use should be documented in the methods or acknowledgments sections (3). Also Elsevier created guidelines on the use of AI-assisted writing for scientific production, confirming the rules imposed by Springer and requiring the authors to specify the AI tools employed, giving details on their use. Elsevier declared to be committed to monitor the development around generative AI and to refine the policy if necessary (52).

The misuse of ChatGPT in scientific research could lead to the production of fake scientific abstracts, papers, and bibliographies. In the earlier versions of ChatGPT (up to the 15th December version), when asked to cite references to support its statements, the output was a list of fake bibliographic references. (e.g., fabricated output reference: *Li, X., & Kim, Y. (2020). Fake academic papers generated by AI: A threat to the integrity of research. PLOS ONE, 15* (3)*, e0231891*.)

The usage of real authors’ names, journals, and plausible titles makes the fake reference difficult to immediately spot. This calls for preventive actions, such as the mandatory use of the digital object identifier system (DOI), which could be used to rapidly and accurately identify fake references.

In fields where fake information can endanger people’s safety, such as medicine, journals may have to take a more rigorous approach to verify the information as accurate (53). A combined evaluation by more up-to-date AI-output detectors and human reviewers is necessary to identify AI-generated scientific abstracts and papers, though this process may be time-consuming and imperfect. We, therefore, suggest adopting a “detectable-by-design” policy: the release of new generative AI models by the tech industry to the public should be permitted only if the output generated by the AI is detectable and thus can be unequivocally identified as AI-produced. The impact that generating false and potentially mystifying texts can have on people’s health is huge. The issue of the dissemination of untruthful information has long been known: starting with the unforgettable Wakefield case and the then-generated disbelief that vaccines can cause autism, (54) to the current observation of non-conservative behavior evidenced by the various phases of the COVID-19 pandemic (55). In this context, it has been more evident than ever that junk and manipulative research, through underperforming studies or with study designs unfit to carry out the intended research objective, has had an impact on the behavior of the general population and, more worryingly, on health professionals (56).

The diffusion of misinformation conveyed through rapidly disseminated channels such as mass media and social networks, can generate the phenomenon known as infodemic (57). The consequence on the scientific framework is considerable, even with implications on possible healthcare choices, already a determining factor in the recent pandemic. (58) Infodemic can influence medical decision-making on treatment or preventive measures, (59, 60) for example some people used hydroxychloroquine as a treatment for COVID-19 based on false or unproven information, endorsed by popular and influential people (61). The risk is that we may face a new health emergency where new information can be rapidly produced using LLMs to generate human-like texts ready to spread even if incorrect or manipulated. The concept of infodemic was introduced in 2003 by Rothkopf as an “epidemic of information,” (62) and evolved in 2020 after the COVID19 pandemic, integrating the element of rapid misinformation spreading (63). With the global diffusion of LLMs the infodemic concept must evolve again into the one of “AI-driven Infodemic.” Not only is it possible to rapidly disseminate misinformation *via* social media platforms and other outlets, but also to produce exponentially growing amounts of health-related information, regardless of one’s knowledge, skills, and intentions. Given the nature of social media content diffusion, LLMs could be used to create content specifically designed for target population groups and in order to go viral and foster misinformation spread. We foresee a scenario in which human-like AI-produced contents will dramatically exacerbate every future health threat that can generate infodemics, that from now on will be AI-driven. Social media and gray literature have already been the ground for infodemic, (63) but scientific literature could become a new and powerful means of disinformation campaigns. The potential of LLMs and in particular ChatGPT in easily generating human-like texts, could convey excessive and, without proper control, low-quality scientific literature production in the health field. The abundance of predatory journals, that accept articles for publication without performing quality checks for issues such as plagiarism or ethical approval, (64) could allow the flooding of the scientific literature with AI-generated articles on an unprecedented scale. The consequences on the integrity of the scientific process and the credibility of the literature would be dreadful (65).

## 6. Discussion

Large language models have already shown hints of their potential in supporting scientific research and in the next months we expect a growing amount of papers talking about the use of ChatGPT in this field.

The accessibility and astonishing abilities of ChatGPT made it popular across the world and allowed it to achieve a milestone, setting AI conversational tools to the next level.

But soon after its release possible threats emerged, ChatGPT’s ability to follow user’s instruction is a double-edged sword: on one hand, this approach makes it great at interacting with humans, on the other hand being submissive *ab origine* exposes it to misuse, for example by generating convincing human-like misinformation.

The field of medical research may be a great source for both opportunities and threats coming from this novel approach.

Given that the scientific community has not yet determined the principles to follow for a helpful and safe use of this disruptive technology, the risks coming from the fraudulent and unethical use of LLMs in the health context cannot be ignored and should be assessed with a proactive approach.

We define the novel concept of “AI-driven infodemic,” a public health threat coming from the use of LLMs to produce a vast amount of scientific articles, fake news, and misinformative contents. The “AI-driven infodemic” is a consequence of the use of LLM’s ability to write large amounts of human-like texts in a short period of time, not only with malicious intent, but in general without any scientific ground and support. Beyond text-based content, other AI tools, such as generative-adversarial networks, could also generate audio and video Deepfakes that could be used to disseminate misinformation content, especially on social media (66). Political Deepfakes already contributed toward generalized indeterminacy and disinformation (67).

To address this public health threat is important to raise awareness and rapidly develop policies through a multidisciplinary effort, updating the current WHO public health research agenda for managing infodemics (68). There is a need for policy action to ensure that the benefits of LLMs are not outweighed by the risks they pose. In this context, we propose the detectable-by-design approach, which involves building LLMs with features that make it easier to detect when they are being used to produce fake news or scientific articles. However, implementing this approach could slow down the development process of LLMs, and for this reason, it might not be readily accepted by AI companies. The constitution of groups of experts inside health international agencies (e.g., WHO, ECDC) dedicated to monitor the use of LLMs for fake news and scientific articles production is needed, as the scenario is rapidly evolving and the AI-driven infodemic threat is forthcoming. Such groups could work closely with AI companies to develop effective strategies for detecting and preventing the use of LLMs for nefarious purposes. Additionally, there might be a need for greater regulation and oversight of the AI industry to ensure that LLMs are developed and used responsibly. Recently, the President of the Italian Data Protection Authority (DPA) has taken action against Open AI for serious breaches of the European legislation on personal data processing and protection (69). The DPA has imposed a temporary ban on ChatGPT in Italy due to the company’s failure to provide adequate privacy information to its users its and lack of a suitable legal basis for data collection. The absence of a suitable legal basis for data collection raises serious concerns about the ethical implications of using personal data without consent or an adequate legal framework.

In the WHO agenda, AI is considered a possible ally to fight infodemics, allowing automatic monitoring for misinformation detection; but the rise of LLMs and in particular ChatGPT should raise concerns that it could play an opposite role in this phenomenon.

LLMs will continue to improve and rapidly become precious allies for researchers, but the scientific community needs to ensure that the advances made possible by ChatGPT and other AI technologies are not overshadowed by the risks they pose. All stakeholders should foster the development and deployment of these technologies aligned with the values and interests of society. It is crucial to increase understanding of AI challenges of transparency, accountability, and fairness in order to develop effective policies. A science-driven debate to develop shared principles and legislation is necessary to shape a future in which AI has a positive impact on public health, not having such a conversation could result in a dangerous AI-fueled future (70).

## Data availability statement

The original contributions presented in the study are included in the article/Supplementary material, further inquiries can be directed to the corresponding author.

## Author contributions

LDA, FB, and GA conceived the paper, performed the literature search, and drafted the manuscript. GP, AT, PF, and CR provided expert insights and contributed to the manuscript revision. LDA and FB contributed equally to the manuscript. All authors contributed to the article and approved the submitted version.

## Funding

This work was supported also by the Italian Ministry of Health with “Current Research funds” for Bambino Gesù Children’s Hospital.

## Conflict of interest

AT received honoraria for education activities on digital health and immunizations from Roche, AstraZeneca, Novartis, Merck, MSD, Pfizer, Sanofi.

The remaining authors declare that the research was conducted in the absence of any commercial or financial relationships that could be construed as a potential conflict of interest.

## Publisher’s note

All claims expressed in this article are solely those of the authors and do not necessarily represent those of their affiliated organizations, or those of the publisher, the editors and the reviewers. Any product that may be evaluated in this article, or claim that may be made by its manufacturer, is not guaranteed or endorsed by the publisher.

## References

[1] ChatGPT: Optimizing language models for dialogue [internet]. Available at: https://openai.com/blog/chatgpt/

[2] Everyone’s having a field day with ChatGPT –But nobody knows how it actually works [internet]. Available at: https://theconversation.com/everyones-having-a-field-day-with-chatgpt-but-nobody-knows-how-it-actually-works-196378

[3] Tools such as ChatGPT threaten transparent science; here are our ground rules for their use. Nature [Internet]. (2023) 613:612. doi: 10.1038/d41586-023-00191-1 36694020

[4] Thorp HH. ChatGPT is fun, but not an author In: Science. New York, N.Y: NLM (Medline) (2023). 379: 313. doi: 10.1126/science.adg7879 36701446

[5] Else H. How a torrent of COVID science changed research publishing -in seven charts. Nature. (2020) 588:553. doi: 10.1038/d41586-020-03564-y, PMID: 33328621

[6] Fraser N Brierley L Dey G Polka JK Pálfy M Nanni F. The evolving role of preprints in the dissemination of COVID-19 research and their impact on the science communication landscape. PLoS Biol [Internet]. (2021) 19:e3000959. doi: 10.1371/journal.pbio.3000959, PMID: 33798194 PMC8046348

[7] Flanagin A Fontanarosa PB Bauchner H. Preprints involving medical research—do the benefits outweigh the challenges? JAMA [Internet]. (2020) 324:1840–3. doi: 10.1001/jama.2020.20674, PMID: 33170226

[8] Cinelli M Quattrociocchi W Galeazzi A Valensise CM Brugnoli E Schmidt AL. The COVID-19 social media infodemic. Sci Rep. (2020) 10:16598. doi: 10.1038/s41598-020-73510-5, PMID: 33024152 PMC7538912

[9] Shams R. Semi-supervised classification for natural language processing. (2014); Available at: https://arxiv.org/abs/1409.7612v1

[10] Vaswani A Shazeer N Parmar N Uszkoreit J Jones L Gomez AN. Attention is all you need. Adv Neural Inf Process Syst [Internet]. (2017) 2017:5999–6009. doi: 10.48550/arXiv.1706.03762

[11] Devlin J Chang MW Lee K Toutanova K. BERT: pre-training of deep bidirectional transformers for language understanding. NAACL HLT 2019–2019 Conference of the North American Chapter of the Association for Computational Linguistics: Human Language Technologies -Proceedings of the Conference [Internet]. (2018); 1:4171–4186. Available at: https://arxiv.org/abs/1810.04805v2

[12] Openai AR Openai KN Openai TS Openai IS. Improving language understanding by generative pre-training; Available at: https://gluebenchmark.com/leaderboard

[13] Brown TB Mann B Ryder N Subbiah M Kaplan J Dhariwal P. Adv Neural Inf Process Syst [Internet]. (2020). doi: 10.48550/arXiv.2005.14165

[14] Ouyang L Wu J Jiang X Almeida D Wainwright CL Mishkin P. Training Language Models to Follow Instructions with Human Feedback In: Advances in Neural Information Processing Systems (2022). doi: 10.48550/arXiv.2203.02155

[15] Korngiebel DM Mooney SD. Considering the possibilities and pitfalls of generative pre-trained transformer 3 (GPT-3) in healthcare delivery. NPJ Digital Medicine. (2021):1–3. 34083689 10.1038/s41746-021-00464-x PMC8175735

[16] Kasirzadeh A Gabriel I. In Conversation with Artificial Intelligence: Aligning Language Models With Human Values. (2022). doi: 10.48550/arXiv.2209.00731

[17] Markov T Zhang C Agarwal S Eloundou T Lee T Adler S. A Holistic Approach to Undesired Content Detection in the Real World. (2022). doi: 10.48550/arXiv.2208.03274

[18] OpenAI. GPT-4 Technical Report. ArXiv [internet] (2023). doi: 10.48550/arXiv.2303.08774

[19] Lee P Bubeck S Petro J. Benefits, limits, and risks of GPT-4 as an AI Chatbot for medicine. N Engl J Med. (2023) 388:1233–9. doi: 10.1056/NEJMsr2214184. 36988602

[20] Bornmann L Haunschild R Mutz R. Growth rates of modern science: a latent piecewise growth curve approach to model publication numbers from established and new literature databases. Hum Soc Sci Commun 2021 8:1 [Internet]. (2021) 8:1–15. doi: 10.1057/s41599-021-00903-w

[21] Extance A. How AI technology can tame the scientific literature. Nature. (2018) 561:273–4. doi: 10.1038/d41586-018-06617-5, PMID: 30202054

[22] Lee J Yoon W Kim S Kim D Kim S So CH. BioBERT: a pre-trained biomedical language representation model for biomedical text mining. Bioinformatics [Internet]. (2020) 36:1234–40. doi: 10.1093/bioinformatics/btz682, PMID: 31501885 PMC7703786

[23] Luo R Sun L Xia Y Qin T Zhang S Poon H. BioGPT: generative pre-trained transformer for biomedical text generation and mining. Brief Bioinform [Internet]. (2022) 23:1–11. doi: 10.1093/bib/bbac409 36156661

[24] Stanford CRFM Introduces PubMedGPT 2.7B [internet]. Available at: https://hai.stanford.edu/news/stanford-crfm-introduces-pubmedgpt-27b

[25] Singhal K Azizi S Tu T Mahdavi SS Wei J Chung HW. Large Language Models Encode Clinical Knowledge. (2022). doi: 10.48550/arXiv.2212.13138 PMC10396962 37438534

[26] Kung T. H. Cheatham M. Medenilla A. Sillos C. De Leon L. Elepaño C.., Performance of ChatGPT on USMLE: potential for AI-assisted medical education using large language models. *medRxiv [Internet]*. (2022); 2022.12.19.22283643. [Epub ahead of preprint] 10.1101/2022.12.19.22283643v2 PMC9931230 36812645

[27] Nori H King N McKinney SM Carignan D Horvitz E. Capabilities of GPT-4 on Medical Challenge Problems [internet]. (2023). doi: 10.48550/arXiv.2303.13375

[28] Ontoum S Chan JH. Automatic text Summarization of COVID-19 Scientific Research Topics using pre-trained Models from Hugging Face. (2022);1–8.

[29] Batra H Jain A Bisht G Srivastava K Bharadwaj M Bajaj D. CoVShorts: news summarization application based on deep NLP transformers for SARS-CoV-2. 9th International Conference on Reliability, Infocom Technologies and Optimization (Trends and Future Directions), ICRITO 2021. (2021).

[30] Raza S Schwartz B Rosella LC. CoQUAD: a COVID-19 question answering dataset system, facilitating research, benchmarking, and practice. BMC Bioinformatics [Internet]. (2022) 23:210–28. doi: 10.1186/s12859-022-04751-6, PMID: 35655148 PMC9160513

[31] Oniani D Wang Y. A qualitative evaluation of language models on automatic question-answering for COVID-19. Proceedings of the 11th ACM International Conference on Bioinformatics, Computational Biology and Health Informatics, BCB 2020 [Internet]. (2020).

[32] Moreno-Garcia CF Jayne C Elyan E Aceves-Martins M. A novel application of machine learning and zero-shot classification methods for automated abstract screening in systematic reviews. Decision Anal J. (2023) 6:100162. doi: 10.1016/j.dajour.2023.100162

[33] Chen M Tworek J Jun H Yuan Q HPO De Pinto Kaplan J. Evaluating Large Language Models Trained on Code. (2021).

[34] Li Y Choi D Chung J Kushman N Schrittwieser J Leblond R. Competition-level code generation with AlphaCode. Science. (1979) [Internet]) 2022:1092–7. doi: 10.1126/science.abq1158 36480631

[35] Castelvecchi D. Are ChatGPT and AlphaCode going to replace programmers? Nature [Internet]. (2022), 4383 doi: 10.1038/d41586-022-04383-z 36481949

[36] Becker BA Denny P Finnie-Ansley J Luxton-Reilly A Prather J Santos EA. (2020). Programming is Hard –Or at Least it used to be: Educational Opportunities and Challenges of AI code Generation; Programming is Hard –Or at Least it used to be: Educational Opportunities and Challenges of AI Code Generation. ArXiv. [internet] doi: 10.48550/arXiv.2212.01020

[37] Gendron Y Andrew J Cooper C. The perils of artificial intelligence in academic publishing. Crit Perspect Account. (2022) 87:102411. doi: 10.1016/j.cpa.2021.102411

[38] Beam AL Drazen JM Kohane IS Leong TY Manrai AK Rubin EJ. Artificial Intelligence in Medicine. N Engl J Med. (2023) 388:1220–1. doi: 10.1056/NEJMe2206291 36988598

[39] Chen Y Eger S. Transformers Go for the LOLs: Generating (Humourous) Titles from Scientific Abstracts end-to-end. (2022).

[40] King MR. A conversation on artificial intelligence, Chatbots, and plagiarism in higher education. Cell Mol Bioeng. (2023) 16:1–2. Available from: https://link.springer.com/article/10.1007/s12195-022-00754-8. PMID: 36660590 10.1007/s12195-022-00754-8 PMC9842816

[41] O’Connor S ChatGPT. Open artificial intelligence platforms in nursing education: tools for academic progress or abuse? Nurse Educ Pract [Internet]. (2023) 66:103537. doi: 10.1016/j.nepr.2022.103537 36549229

[42] ChatGPT Generative Pre-trained Transformer Zhavoronkov A. Rapamycin in the context of Pascal’s wager: generative pre-trained transformer perspective. Onco Targets Ther. (2022) 9:82–4. doi: 10.18632/oncoscience.571 PMC9796173 36589923

[43] EAM Van Dis Bollen J Van Rooij R Zuidema W Bockting CL. ChatGPT: Five Priorities for Research. 614, 224–226. 10.1038/d41586-023-00288-7 36737653

[44] OpenAI’s new chatbot is multi-talented but still easily tricked. The Verge [Internet]. Available at: https://www.theverge.com/23488017/openai-chatbot-chatgpt-ai-examples-web-demo

[45] Gupta A Singhal A Mahajan A Jolly A Kumar S. Empirical framework for automatic detection of neural and human authored fake news. Proceedings -2022 6th International Conference on Intelligent Computing and Control Systems, ICICCS 2022. (2022);1625–1633.

[46] McGuffie K Newhouse A. The Radicalization Risks of GPT-3 and Advanced Neural Language Models. (2020). doi: 10.48550/arXiv.2009.06807

[47] Sear RF Leahy R Restrepo NJ Lupu Y Johnson NF. Machine learning language models: Achilles heel for social media platforms and a possible solution. AAIML. (2021) 01:191–202. doi: 10.54364/AAIML.2021.1112

[48] Jwa H Oh D Park K Kang JM Lim H. exBAKE: automatic fake news detection model based on bidirectional encoder representations from transformers (BERT). Appl Sci. (2019) 9:4062. doi: 10.3390/app9194062

[49] Keya AJ Wadud MAH Mridha MF Alatiyyah M Hamid MA. AugFake-BERT: handling imbalance through augmentation of fake news using BERT to enhance the performance of fake news classification. Appl Sci. (2022) 12:8398. doi: 10.3390/app12178398

[50] Can AI. Detectors save us from ChatGPT? In: I tried 3 Online Tools to Find Out | ZDNET [internet]. Available at: https://www.zdnet.com/article/can-ai-detectors-save-us-from-chatgpt-i-tried-3-online-tools-to-find-out/

[51] Gordijn B Have H. Ten. ChatGPT: evolution or revolution? Med health care Philos [internet]. (2023) 26:1–2. doi: 10.1007/s11019-023-10136-0 36656495

[52] Elsevier declaration of generative AI in scientific writing [internet]. Available at: https://www.elsevier.com/journals/artificial-intelligence/0004-3702/guide-for-authors

[53] Else H. Abstracts written by ChatGPT fool scientists. Nat Cell Biol. (2023) 613:423. doi: 10.1038/d41586-023-00056-7 36635510

[54] Hopkins J York TN. Wakefield admits fabricating events when he took children’s blood samples. BMJ [Internet]. (2008) 336:850.1–850. doi: 10.1136/bmj.39553.506597.DB PMC2323045 18420676

[55] McBride E Arden MA Chater A Chilcot J. The impact of COVID-19 on health behaviour, well-being, and long-term physical health. Br J Health Psychol [Internet]. (2021) 26:259–70. doi: 10.1111/bjhp.12520, PMID: 33787000 PMC8250322

[56] Casigliani V de Nard F de Vita E Arzilli G Grosso FM Quattrone F. Too much information, too little evidence: is waste in research fuelling the covid-19 infodemic? BMJ [Internet]. (2020) 370:1847. doi: 10.1136/bmj.m2672 32631897

[57] The Lancet Infectious Diseases. The COVID-19 infodemic. Lancet Infect Dis. (2020) 20:875. doi: 10.1016/S1473-3099(20)30565-X, PMID: 32687807 PMC7367666

[58] Tuccori M Convertino I Ferraro S Cappello E Valdiserra G Focosi D. The impact of the COVID-19 “Infodemic” on drug-utilization behaviors: implications for Pharmacovigilance. Drug Saf [Internet]. (2020) 43:699–709. doi: 10.1007/s40264-020-00965-w, PMID: 32572842 PMC7307939

[59] Zarocostas J. How to fight an infodemic. Lancet. (2020) 395:676. doi: 10.1016/S0140-6736(20)30461-X, PMID: 32113495 PMC7133615

[60] Briand SC Cinelli M Nguyen T Lewis R Prybylski D Valensise CM. Infodemics: a new challenge for public health. Cells. (2021) 184:6010–4. doi: 10.1016/j.cell.2021.10.031, PMID: 34890548 PMC8656270

[61] Schwartz IS Boulware DR Lee TC. Hydroxychloroquine for COVID19: the curtains close on a comedy of errors. Lancet Reg Health -Americas. (2022) 11:100268. doi: 10.1016/j.lana.2022.100268, PMID: 35531052 PMC9069223

[62] Rothkopf DJ. When the buzz bites Back. Washington Post[Internet] (2003); Available at: https://www.washingtonpost.com/archive/opinions/2003/05/11/when-the-buzz-bites-back/bc8cd84f-cab6-4648-bf58-0277261af6cd/

[63] Gisondi MA Barber R Faust JS Raja A Strehlow MC Westafer LM. A deadly Infodemic: social media and the power of COVID-19 misinformation. J Med Internet Res. (2022) 24:e35552. doi: 10.2196/35552, PMID: 35007204 PMC8812140

[64] Grudniewicz A Moher D Cobey KD Bryson GL Cukier S Allen K. Predatory journals: no definition, no defence. Nature. (2019) 576:210–2. doi: 10.1038/d41586-019-03759-y, PMID: 31827288

[65] Flanagin A Bibbins-Domingo K Berkwits M Christiansen SL. Nonhuman “authors” and implications for the integrity of scientific publication and medical knowledge. JAMA [Internet]. (2023) 329:637–9. doi: 10.1001/jama.2023.1344, PMID: 36719674

[66] Langguth J Pogorelov K Brenner S Filkuková P Schroeder DT. Don’t trust your eyes: image manipulation in the age of DeepFakes In: Frontiers in Communication. Lausanne: Frontiers Media S.A. (2021).

[67] Vaccari C Chadwick A. Deepfakes and disinformation: exploring the impact of synthetic political video on deception, uncertainty, and trust in news. Social Media+Society. (2020) 6:205630512090340. doi: 10.1177/2056305120903408

[68] WHO Public Health Research Agenda for Managing Infodemics [internet]. Available at: https://www.who.int/publications/i/item/9789240019508

[69] ChatGPT banned in Italy over privacy concerns. BBC news [internet]. Available at: https://www.bbc.com/news/technology-65139406

[70] Vinuesa R Azizpour H Leite I Balaam M Dignum V Domisch S. The role of artificial intelligence in achieving the sustainable development goals. Nat Commun [Internet]. (2020) 11:1–10. doi: 10.1038/s41467-019-14108-y 31932590 PMC6957485

