# Supplementary material for: ChatGPT and the rise of large language models: the new AI-driven infodemic threat in public health
Source: Front Public Health. 2023 Apr 25;11:1166120. doi: 10.3389/fpubh.2023.1166120 (PMC10166793; doi:10.3389/fpubh.2023.1166120)
Supplement: Supplementary file 1 [file Table_1.DOCX]

**Supplementary Table. List of papers where ChatGPT’s role in scientific and medical research is discussed**

|  | **Title** | **Publication date** | **Journal/platform** | **Type of Article** | **DOI/link** |
| --- | --- | --- | --- | --- | --- |
| 1 | Should Using an AI Text Generator to Produce Academic Writing Be Plagiarism? | 03/12/2022 | SSRN | Article | <https://ssrn.com/abstract=4292283> |
| 2 | The Role of AI in Drug Discovery: Challenges, Opportunities, and Strategies | 08/12/2022 | arXiv | Article | <https://doi.org/10.48550/arXiv.2212.08104> |
| 3 | Paraphrase Identification with Deep Learning: A Review of Datasets and Methods | 13/12/2022 | arXiv | Review | <https://doi.org/10.48550/arXiv.2212.06933> |
| 4 | Co-authoring with an AI? Ethical Dilemmas and Artificial Intelligence | 15/12/2022 | SSRN | Article | <https://dx.doi.org/10.2139/ssrn.4303959> |
| 5 | Open artificial intelligence platforms in nursing education: Tools for academic progress or abuse? | 16/12/2022 | Nurse Education in Practice | Article | <https://doi.org/10.1016/j.nepr.2022.103537> |
| 6 | Transformers Go for the LOLs: Generating (Humourous) Titles from Scientific Abstracts End-to-End | 20/12/2022 | arXiv | Article | <https://arxiv.org/abs/2212.10522> |
| 7 | OpenAI ChatGPT Generated Literature Review: Digital Twin in Healthcare | 21/12/2022 | SSRN | Article | <https://dx.doi.org/10.2139/ssrn.4308687> |
| 8 | Rapamycin in the context of Pascal's Wager: generative pre-trained transformer perspective | 21/12/2022 | Oncoscience | Article | <https://doi.org/10.18632/oncoscience.571> |
| 9 | Performance of ChatGPT on USMLE: Potential for AI-Assisted Medical Education Using Large Language Models | 21/12/2022 | medRxiv | Article | <https://doi.org/10.1101/2022.12.19.22283643> |
| 10 | Concerns About the Potential Risks of Artificial Intelligence in Manuscript Writing. Letter. | 23/12/2022 | Journal of Urology | Letter | <https://doi.org/10.1097/JU.0000000000003131> |
| 11 | Did a Robot Write This Title? Creativity, Ownership, Justice, and Copyright Law | 27/12/2022 | SSRN | Article | <https://dx.doi.org/10.2139/ssrn.4304470> |
| 12 | Comparing scientific abstracts generated by ChatGPT to original abstracts using an artificial intelligence output detector, plagiarism detector, and blinded human reviewers | 27/12/2022 | bioRxiv | Article | <https://doi.org/10.1101/2022.12.23.521610> |
| 13 | A Conversation on Artificial Intelligence, Chatbots, and Plagiarism in Higher Education | 02/01/2023 | Cellular and Molecular Bioengineering | Editorial | <https://doi.org/10.1007/s12195-022-00754-8> |
| 14 | AI et al.: Machines Are About to Change Scientific Publishing Forever | 04/01/2023 | ACS Energy Lett. | Letter | <https://doi.org/10.1021/acsenergylett.2c02828> |
| 15 | A conversation with ChatGPT on the role of computational systems biology in stem cell research | 10/01/2023 | Stem cell reports | Editorial | <https://doi.org/10.1016/j.stemcr.2022.12.009> |
| 16 | Abstracts written by ChatGPT fool scientists | 12/01/2023 | Nature | Article | <https://doi.org/10.1038/d41586-023-00056-7> |
| 17 | ChatGPT listed as author on research papers: many scientists disapprove | 18/01/2023 | Nature | Article | <https://doi.org/10.1038/d41586-023-00107-z> |
| 18 | ChatGPT: evolution or revolution? | 19/01/2023 | Med Health Care and Philos | Editorial | <https://doi.org/10.1007/s11019-023-10136-0> |
| 19 | Chatgpt's Scientific Writings: A Case Study on Traffic Safety | 20/01/2023 | SSRN | Article | <https://ssrn.com/abstract=4329120> |
| 20 | Putting ChatGPT’s Medical Advice to the (Turing) Test | 24/01/2023 | medRxiv | Article | <https://doi.org/10.1101/2023.01.23.23284735> |
| 21 | Chatting about ChatGPT: How May AI and GPT Impact Academia and Libraries? | 24/01/2023 | SSRN | Article | <https://ssrn.com/abstract=4333415> |
| 22 | Tools such as ChatGPT threaten transparent science; here are our ground rules for their use | 24/01/2023 | Nature | Editorial | <https://doi.org/10.1038/d41586-023-00191-1> |
| 23 | ChatGPT is fun, but not an author | 26/01/2023 | Science | Editorial | <https://doi.org/10.1126/science.adg7879> |
| 24 | ChatGPT and Other Large Language Models Are Double-edged Swords | 26/01/2023 | Radiology | Editorial | <https://doi.org/10.1148/radiol.230163> |
| 25 | A Computer Wrote this Paper: What ChatGPT Means for Education, Research, and Writing | 27/01/2023 | SSRN | Article | <https://dx.doi.org/10.2139/ssrn.4338981> |
| 26 | Feasibility Study on Utilization of the Artificial Intelligence GPT-3 in Public Health | 28/01/2023 | Preprints.org | Article | <http://dx.doi.org/10.1016/S0140-6736(19)32380-3> |
| 27 | How Will Artificial Intelligence Affect Scientific Writing, Reviewing and Editing? The Future is Here… | 31/01/2023 | Arthroscopy: The Journal of Arthroscopic and Related Surgery | Letter | <https://doi.org/10.1016/j.arthro.2023.01.014> |
| 28 | ChatGPT, An Artificial Intelligence Chatbot, Is Impacting Medical Literature | 31/01/2023 | Arthroscopy: The Journal of Arthroscopic and Related Surgery | Letter | <https://doi.org/10.1016/j.arthro.2023.01.015> |
| 29 | Nonhuman “Authors” and Implications for the Integrity of Scientific Publication and Medical Knowledge | 31/01/2023 | JAMA | Editorial | <https://doi.org/10.1001/jama.2023.1344> |
| 30 | ChatGPT and the Future of Medical Writing | 02/02/2023 | Radiology | Comment | <https://doi.org/10.1148/radiol.223312> |
| 31 | ChatGPT Is Shaping the Future of Medical Writing but Still Requires Human Judgment | 02/02/2023 | Radiology | Editorial | <https://doi.org/10.1148/radiol.230171> |
| 32 | ChatGPT: five priorities for research  Conversational AI is a game-changer for science. Here’s how to respond. | 03/02/2023 | Nature | Comment | <https://doi.org/10.1038/d41586-023-00288-7> |
| 33 | Daily briefing: Science urgently needs a plan for ChatGPT | 03/02/2023 | Nature | Article | <https://doi.org/10.1038/d41586-023-00360-2> |
| 34 | What ChatGPT and generative AI mean for science  Researchers are excited but apprehensive about the latest advances in artificial intelligence. | 06/02/2023 | Nature | Article | <https://doi.org/10.1038/d41586-023-00340-6> |
| 35 | Generating scholarly content with ChatGPT: ethical challenges for medical publishing | 06/02/2023 | The Lancet Digital Health | Comment | https://doi.org/10.1016/S2589-7500(23)00019-5 |
| 36 | Letter to Editor: NLP systems such as ChatGPT cannot be listed as an author because these cannot fulfill widely adopted authorship criteria | 07/02/2023 | Accountability in Research | Letter | <https://doi.org/10.1080/08989621.2023.2177160> |
